# Is Preoperative Serum Bicarbonate an Undervalued Risk Factor in Cardiac Surgery?

**DOI:** 10.1007/s12265-026-10791-7

**Published:** 2026-07-13

**Authors:** Wesley Chorney, John Hinchion

**Affiliations:** 1 College of Medicine and Health, University College Cork, College Road, Cork, T12K8AF County Cork Ireland; 2 Department of Cardiothoracic Surgery, Cork University Hospital, Cork, T12DFK4 County Cork Ireland

**Keywords:** Cardiothoracic surgery, Bicarbonate, Mortality, Regression modeling

## Abstract

**Abstract:**

Existing risk stratification methods in cardiothoracic surgery do not use pre-operative bicarbonate levels. We investigate if these levels carry predictive value when compared with demographic, hematologic, and other renal function indicators. We used the MIMIC-IV database to identify 11,261 patients on the cardiothoracic surgery unit who underwent ICD-9 or ICD-10 coded cardiac procedures. 30-day mortality was then modeled with respect to these indicators. Minimum pre-operative bicarbonate level is an independent risk factor for 30-day mortality post-cardiothoracic surgery ($p=0.005$). The lowest quartile with respect to bicarbonate had increased mortality compared to all other quartiles, suggesting that patients may be classified into high- or low-risk based on bicarbonate lesser or greater than 20 mmol/L. Minimum pre-operative bicarbonate levels carry predictive information with respect to post-operative mortality. Bicarbonate could be considered alongside existing risk stratification methods in addition to other renal function indicators to improve risk stratification.

**Graphical abstract:**

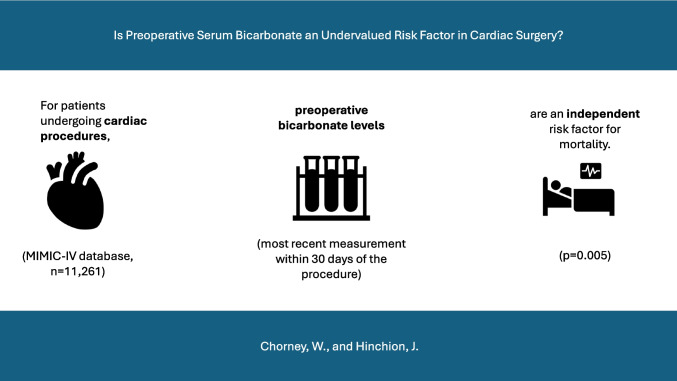

## Introduction

Surgery incurs significant trauma to the body, and thus several risks are involved. Cardiothoracic surgeries are complex operations, with notable mortality rates. For instance, estimates of mortality due to abdominal aortic aneurysm (AAA) repair range from 2.9–10.4% based on whether patients have complications [1]. Mortality also varies based on the type of surgery, the surgeon, and the location [2, 3]. Due to significant variations in post-surgery mortality, which are dependent on a number of factors, risk scores have been developed to provide evidence-based estimates of mortality. With respect to cardiothoracic surgery, the EuroSCORE II and STS score are commonly used [4, 5].

The EuroSCORE II and STS Score involve factors such as operation type, cardiovascular and pulmonary morbidity, and renal status (as measured by creatinine levels or creatinine clearance). However, other indicators of renal function may provide additional benefit in mortality prediction. Indeed, recent work has shown that bicarbonate may be a useful biomarker [6], and given the link between metabolic acidosis and mortality [7], this is unsurprising. Despite the link between acidosis and mortality, administering preoperative bicarbonate has not shown a statistically significant improvement in renal function or acute kidney injury (AKI) post-cardiothoracic surgery [8, 9], with some evidence that it may even prolong intensive care unit (ICU) stay [10].

While the link between bicarbonate levels and short-term postoperative outcomes has been investigated for colorectal cancer, where low bicarbonate levels were not found to predict outcomes (in contrast to lactate levels) [11], overall, a paucity of studies on the subject have been conducted. The poor prognostic implications of hyperlactatemic metabolic acidosis have been established [12], and with respect to type A aortic dissection repair, a univariate analysis determined the lowest preoperative bicarbonate level was associated with operative mortality [13]. In addition, low preoperative bicarbonate was also associated with AKI after cardiac surgery [14]. Expanding on these findings, we provide evidence that low preoperative bicarbonate is a poor prognostic factor generally with respect to mortality post-cardiothoracic surgery and across many different cardiothoracic procedures.

## Methods

### Dataset

Patients are drawn from the MIMIC-IV dataset [15, 16], available freely from PhysioNet [17]. The MIMIC-IV dataset is comprised of de-identified data from 364,627 patients who either visited the emergency room or were admitted to the ICU at Beth Israel Deaconess Medical Center in Boston. Comprehensive data from over 546,028 hospitalizations and 94,458 ICU stays between 2008 and 2022 is included, with data such as demographics, vital signs, laboratory tests, medications, procedures, diagnoses, and clinical notes. Patients selected for inclusion had undergone a cardiothoracic procedure as defined by ICD 9 codes with the prefix 35–38 and ICD 10 codes with the prefix 02–06.

For each included patient, patient demographics, including age, sex, blood pressure, and BMI were recorded. In addition, standard renal function indicators, including minimum and maximum pre-operative calcium, chloride, creatinine, glucose, lactate, magnesium, phosphate, potassium, sodium, urea nitrogen, and pH were taken, as well as maximum and minimum preoperative bicarbonate, as in [6, 13]. Herein, we note a limitation of the study in that due to the nature of the database, it was not possible to extract data allowing for the calculation of either the STS Score or the EuroSCORE II for each patient.

### Statistical Analysis

All statistical analyses were performed with the *statsmodels* package using Python 3.10. Comparisons of continuous variables between groups were made with Welch’s two-sided *t*-test, with a significance level of $\alpha =0.05$. Categorical variables were compared with a chi-square test. Descriptive statistics were computed for preoperative variables, with groups defined by 30-day mortality. In order to predict 30-day mortality, a stepwise multivariable logistic regression model was used. A significance level of 0.03 was required for the addition of another variable in the model, and a significance level of 0.05 was required for a variable to remain in the model. Regression coefficients are estimated conditional on the presence of other predictors to adjust for co-linearity and potential confounding between predictors, and the likelihood ratio test was used to quantify the significance of each variable, with respect to other predictors, in the final model.

## Results

Table 1 gives an overview of the values in the dataset, partitioned by patient mortality.

Across all variables, multiple statistically significant differences were observed between deceased and surviving patients. Notably, deceased patients had substantially lower minimum preoperative bicarbonate levels ($17.17 \pm 5.64$ mmol/L) compared to survivors ($21.90 \pm 3.98$ mmol/L), with a mean difference of 4.73 mmol/L. This marked disparity supports the hypothesis that lower bicarbonate levels are associated with worse outcomes.

Patients who died also had significantly higher levels of several biochemical markers associated with metabolic dysregulation. For instance, maximum preoperative lactate levels were markedly elevated in deceased patients ($7.07 \pm 5.55$ mmol/L versus $2.58 \pm 1.70$ mmol/L in survivors), indicating a strong association between preoperative lactic acidosis and mortality. Similar trends were noted for phosphate, creatinine, glucose, and urea nitrogen, all of which were elevated in the deceased cohort, reflecting widespread renal and metabolic compromise.

**Table 1:** Distribution of values in the dataset, partitioned by patient mortality. Results are reported as means plus or minus standard deviations. *p*-values are obtained via Welch’s independent two-sample *t*-tests

Variable	No Mortality	Mortality	*p*-value
Age	65.91 ± 11.84	67.07 ± 14.02	0.073
Systolic Blood Pressure	133.53 ± 19.04	128.84 ± 20.19	$<0.001$
Diastolic Blood Pressure	75.12 ± 12.53	71.91 ± 12.55	$<0.001$
BMI	28.62 ± 6.09	26.00 ± 4.88	0.074
Minimum Preoperative Bicarbonate	22.24 ± 2.89	17.39 ± 5.33	$<0.001$
Maximum Preoperative Bicarbonate	26.74 ± 3.36	26.82 ± 5.42	0.764
Minimum Preoperative Calcium	8.56 ± 0.77	7.87 ± 0.97	$<0.001$
Maximum Preoperative Calcium	9.17 ± 0.73	9.57 ± 1.68	$<0.001$
Minimum Preoperative Chloride	100.94 ± 4.94	95.88 ± 6.98	$<0.001$
Maximum Preoperative Chloride	108.17 ± 4.36	106.29 ± 7.19	$<0.001$
Minimum Preoperative Creatinine	0.99 ± 0.82	1.66 ± 1.42	$<0.001$
Maximum Preoperative Creatinine	1.37 ± 1.32	3.20 ± 2.38	$<0.001$
Minimum Preoperative Glucose	97.65 ± 25.95	92.68 ± 51.91	0.037
Maximum Preoperative Glucose	211.86 ± 149.27	268.03 ± 175.95	$<0.001$
Minimum Preoperative Lactate	1.28 ± 0.48	1.89 ± 2.11	$<0.001$
Maximum Preoperative Lactate	2.87 ± 1.54	7.49 ± 5.43	$<0.001$
Minimum Preoperative Magnesium	1.97 ± 0.32	1.84 ± 0.33	$<0.001$
Maximum Preoperative Magnesium	2.37 ± 0.59	2.71 ± 0.62	$<0.001$
Minimum Preoperative Phosphate	3.18 ± 0.85	3.23 ± 1.56	0.493
Maximum Preoperative Phosphate	4.17 ± 1.25	6.36 ± 2.27	$<0.001$
Minimum Preoperative Potassium	3.91 ± 0.46	3.67 ± 0.65	$<0.001$
Maximum Preoperative Potassium	4.66 ± 0.67	5.36 ± 0.97	$<0.001$
Minimum Preoperative Sodium	137.10 ± 3.90	133.23 ± 6.06	$<0.001$
Maximum Preoperative Sodium	140.73 ± 3.18	142.52 ± 6.48	$<0.001$
Minimum Preoperative Urea Nitrogen	17.20 ± 10.72	28.01 ± 20.11	$<0.001$
Maximum Preoperative Urea Nitrogen	25.96 ± 18.90	56.44 ± 33.64	$<0.001$
Minimum Preoperative pH	6.25 ± 0.73	6.10 ± 0.83	$<0.001$
Maximum Preoperative pH	7.41 ± 0.32	7.43 ± 0.27	0.042

Age and BMI are generally associated with mortality; however, in the dataset in question, the association is not statistically significant. In the dataset, values such as blood pressure are also clearly correlated with mortality. Deceased patients had lower systolic and diastolic pressures on average, even though values fluctuated significantly between groups, made evident by the large standard deviations. The decreased values could potentially be indicative of overall frailty or cardiovascular instability, though some co-linearity most likely exists given that not all of these demographic variables were included in the model.

**Table 2:** Coefficients of the multistep logistic regression model. The *y*-intercept is omitted for clarity

Variable	Coefficient	Standard Error	Z Score	P Value	95% Confidence Interval
Maximum Phosphate	0.3114	0.059	5.256	0.000	(0.195, 0.428)
Maximum Lactate	0.3125	0.031	9.983	0.000	(0.251, 0.374)
Maximum Urea Nitrogen	0.0111	0.004	2.729	0.006	(0.003, 0.019)
Maximum Chloride	−0.0257	0.007	−3.933	0.000	$(-0.038, -0.013)$
Diastolic Blood Pressure	0.0063	0.002	2.880	0.004	(0.002, 0.011)
Minimum Bicarbonate	−0.0608	0.021	−2.837	0.005	$(-0.103, -0.019)$
Maximum Calcium	0.1189	0.038	3.147	0.002	(0.045, 0.193)
Minimum Magnesium	−0.5341	0.177	−3.013	0.003	$(-0.881, -0.187)$
Minimum Urea Nitrogen	0.0181	0.006	2.800	0.005	(0.005, 0.031)
Previous Operations	0.0308	0.012	2.603	0.009	(0.008, 0.054)
Age	0.0172	0.007	2.625	0.009	(0.004, 0.030)
Minimum pH	0.1625	0.064	2.541	0.011	(0.037, 0.288)

Table 2 shows the coefficients and associated statistics from the final logistic regression model used to predict 30-day mortality. Several variables were identified as independent predictors^1^ of mortality. Most notably, minimum bicarbonate remained a significant predictor after adjusting for other covariates ($\text {coefficient}= -0.0608$, $p = 0.005$), confirming its utility as a prognostic biomarker. The negative coefficient indicates that lower bicarbonate levels were associated with increased mortality risk.

Other independent predictors of mortality included maximum lactate ($\text {coefficient} = 0.3125$, $p < 0.001$), maximum phosphate ($\text {coefficient} = 0.3114$, $p < 0.001$), and both maximum and minimum urea nitrogen ($\text {coefficient} = 0.0111$, $p = 0.006$ and $\text {coefficient} = 0.0181$, $p = 0.005$), further underscoring the prognostic importance of metabolic and renal dysfunction. Notably, minimum and maximum urea nitrogen are both significant predictive factors for mortality. This could be due to the fact that a high minimum urea nitrogen signifies persistent renal dysfunction, while a high maximum urea nitrogen signifies a significant episode of renal dysfunction.

**Fig. 1:**
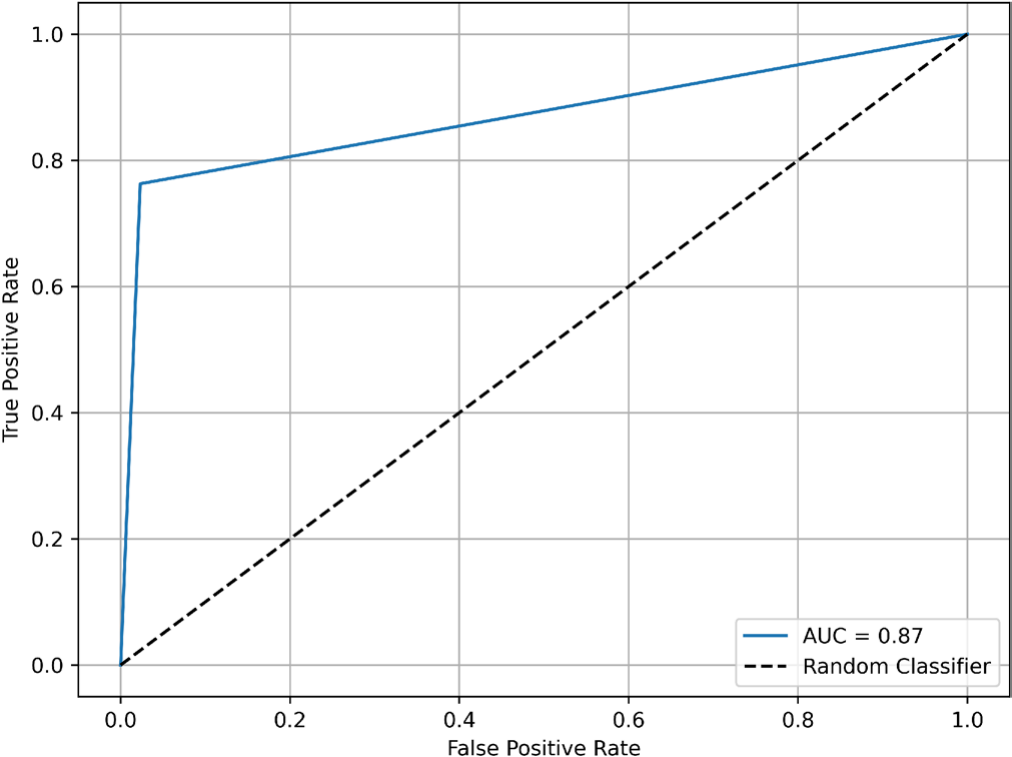
Area under the receiver-operator characteristic curve for the logistic regression model, compared to a classifier that produces random guesses

**Fig. 2:**
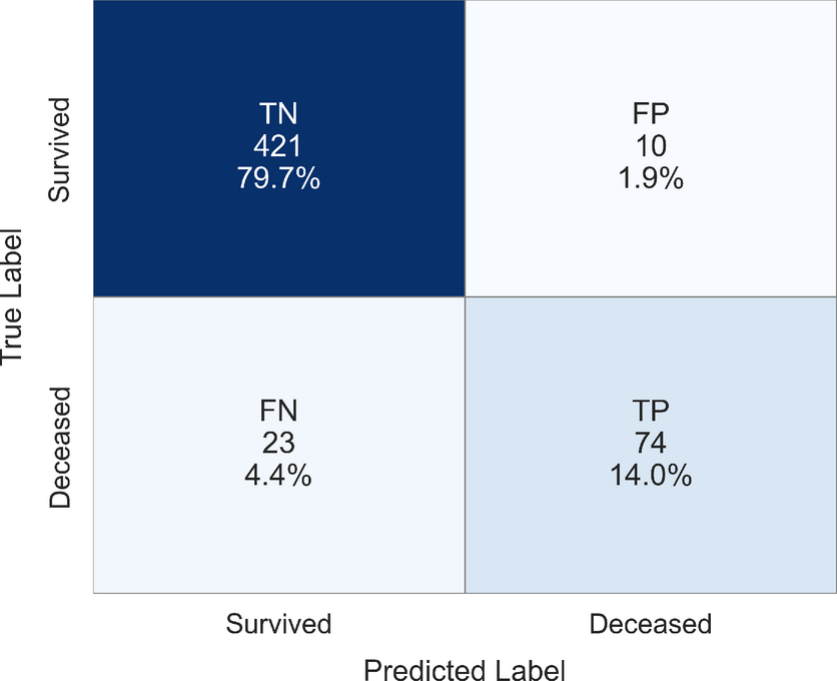
Confusion matrix for the logistic regression model

Figure 1 displays the area under the receiver-operator characteristic curve (AUC-ROC) of the resultant model, while Fig. 2 displays a confusion matrix for the model. We use *TP*, *TN*, *FP*, *FN* to denote true positives, true negatives, false positives, and false negatives, respectively. While the goal of this study was focused on investigating whether bicarbonate was a relevant risk factor for cardiothoracic surgery, the resultant logistic regression model achieves adequate discrimination between survival and mortality, achieving a $93.75\%$ accuracy, $76.29\%$ sensitivity, and $97.68\%$ specificity, with an AUC-ROC of 0.87 (a value of 1 would correspond to a perfect classifier). Given the high specificity, the model could potentially be used to help to identify high risk surgical cases, so that further pre-operative optimization could be undertaken.

**Fig. 3:**
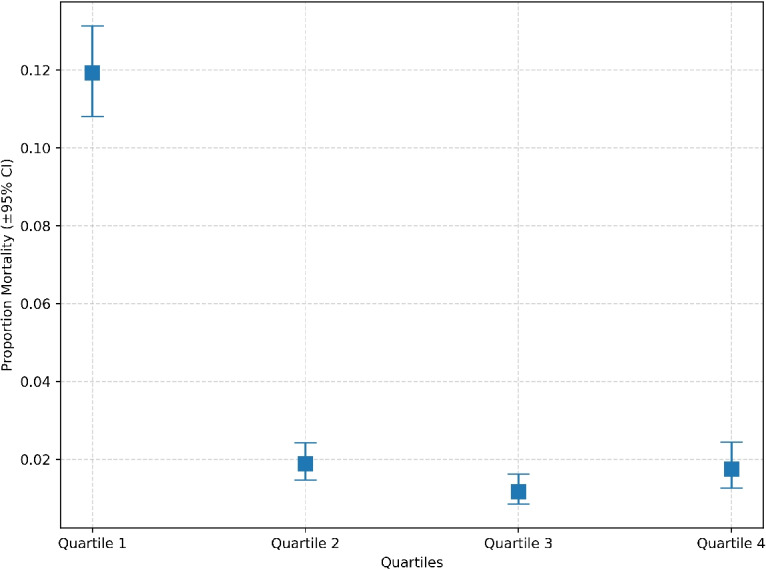
Proportional mortality by minimum pre-operative bicarbonate level quartiles, with 95% confidence intervals according to the Wilson interval formula for proportions

Finally, Fig. 3 shows the proportion mortality of all patients in the dataset by minimum pre-operative bicarbonate level quartiles, with error bars representing 95% confidence intervals according to the Wilson interval formula for proportions. For those patients in the lowest quartile, mortality is significantly higher than any other quartile; whereas no statistically significant difference for patients in the second, third, and fourth quartile was observed.

## Discussion

While the impact of pre-operative minimum bicarbonate levels are insufficiently studied, the results we obtained are well-supported by and confirmatory of the literature for other risk factors. For instance, elevated pre-operative phosphate levels are known to increase risk for vascular calcification and have been shown to be an independent risk factor for mortality post cardiothoracic surgery [18]. Given that lactic acidosis arises in settings such as hypoxia and tissue ischemia, it is perhaps unsurprising that elevated pre-operative lactic acidosis is associated with increased mortality post-surgery [19, 20]. Both elevated and reduced blood urea nitrogen, elevated chloride, calcium, and decreased magnesium are potential markers for renal dysfunction, which can greatly impact post-surgical outcomes. While there is an established link between blood urea nitrogen and poorer post-cardiac surgery outcomes [21], the effect of hyperchloremia is less well-studied. Hyperchloremia is known to be an independent risk factor for mortality in non-cardiac surgery [22] and is known to be associated with poorer renal outcomes post-cardiothoracic surgery [23], but the current study confirms that it is another independent risk factor for mortality post-cardiothoracic surgery.

While pre-operative bicarbonate levels are not typically included in risk stratification for cardiothoracic surgery, there is a salient link between metabolic acidosis and various organ dysfunction that is a likely explanation for why it is an independent risk factor. For instance, metabolic acidosis leads to an increase in hydrogen ions, which compete with calcium to bind troponin C, ultimately reducing myocardial contractility, while also causing vasodilation and reducing the sensitivity to catecholamines [24]. In conjunction with cardiothoracic surgery, which is itself a significant stressor to the cardiovascular system, these impediments to normal function may contribute to poor outcomes. Furthermore, metabolic acidosis also impairs liver and immune function, which could additionally contribute to poorer outcomes post-cardiothoracic surgery [25, 26].

It is worthwhile to note that the significance of low pre-operative bicarbonate levels was obtained while also considering elevated or decreased lactic acid and chloride levels (as well as other renal indicators). In particular, given that both lactic acid and bicarbonate have some relationship in metabolic acidosis, that minimum pre-operative bicarbonate is an independent risk factor indicates that it reflects unique aspects of metabolic dysfunction which is not, or is insufficiently, captured by lactic acid levels alone. Furthermore, given that bicarbonate remained an independent risk factor despite inclusion of typical renal function indicators such as creatinine and blood urea nitrogen, it is possible that minimum bicarbonate levels are reflective of a physiological impairment beyond renal function alone.

Figure 3 also suggests a non-linear relationship between bicarbonate and pre-operative mortality. In particular, given that the lowest quartile group experienced significantly higher mortality across all procedures, while the remaining quartiles were similar, this suggests a simple heuristic rule in gauging pre-operative risk with respect to minimum pre-operative bicarbonate levels. In the given datset, the boundary between first and second quartile was given by a minimum pre-operative bicarbonate level of 20 mmol/L. Therefore, patients can be stratified into high (minimum pre-operative bicarbonate levels less than 20 mmol/L) or low (minimum pre-operative bicarbonate levels greater than or equal to 20 mmol/L) with respect to bicarbonate levels alone. Of course, such a heuristic should never supersede established risk stratification methods, but may offer an additional insight into patient risk.

Despite the strengths of the study, several limitations warrant consideration. First, the use of retrospective data from the MIMIC-IV database limits generalizability, particularly to non-U.S. or non-ICU populations. Second, the inability to compute standardized surgical risk scores such as the EuroSCORE II or STS score due to data constraints restricts direct comparisons between our model and established methods of risk stratification. Third, residual confounding due to unmeasured variables, such as surgical complexity, fluid status, or intraoperative events, cannot be excluded.

Future studies should seek to validate these findings prospectively and evaluate whether interventions targeting acid-base balance and the optimization of bicarbonate levels preoperatively could improve patient outcomes. Finally, incorporation of pre-operative bicarbonate levels should also be considered in future risk stratification methods. Pre-operative levels could also be used in adjunct models which could then be combined with existing methods of risk stratification, potentially leading to an increased overall performance.

## Conclusion

This study demonstrates that low pre-operative bicarbonate levels are an independent predictor of 30-day mortality following cardiothoracic surgery. After adjustment for renal, metabolic, and hemodynamic variables, minimum pre-operative bicarbonate remained significantly associated with increased mortality risk, reinforcing the potential value of incorporating this measurement into pre-operative risk assessments.

The findings are in agreement with studies that show metabolic acidosis plays a critical role in determining patient outcomes, and additionally support that multifactorial mechanisms involving cardiovascular, renal, hepatic, and immune system compromise may be captured in part by bicarbonate levels. These results are consistent with prior literature emphasizing the importance of biochemical markers such as lactate and phosphate, but highlight that bicarbonate, which is an easily obtainable and inexpensive lab value, can serve as a practical adjunct in risk stratification methods.

While existing models such as the EuroSCORE II and STS score do not currently include minimum pre-operative bicarbonate as a variable, our findings suggest that it could enhance predictive accuracy, especially in settings where full scoring systems are unavailable. Future work may focus on validating these findings across different patient populations and surgical contexts.

In conclusion, pre-operative bicarbonate levels should be considered in the pre-operative evaluation of cardiothoracic patients, particularly for identifying individuals at heightened risk of post-operative mortality who may benefit from additional optimization or closer post-operative monitoring.

## Data Availability

Data used in this work is freely available via PhysioNet.

## Footnotes

1 We refer to the independent variables selected for inclusion into the logistic regression model as “predictors," inasmuch as it is a standard term for variables in a regression model, and not to imply any causality.
